# Percutaneous Management of Bioprosthetic Mitral Valve Dehiscence with Combined Valve-in-Valve Replacement and Paravalvular Leak Closure

**DOI:** 10.14797/mdcvj.1140

**Published:** 2022-12-08

**Authors:** Hani Alturkmani, Jack Xu, Adib Chaus, Srikanth Vallurupalli, Gaurav Dhar

**Affiliations:** 1 University of Arkansas for Medical Sciences, Little Rock, Arkansas, US; 2 Central Arkansas Veterans Health System, Little Rock, Arkansas, US

**Keywords:** mitral valve endocarditis, valvular dehiscence, valve in valve, paravalvular leak

## Abstract

This case report describes a patient with bioprosthetic mitral valve dehiscence that resulted in severe paravalvular regurgitation and cardiogenic shock. Due to prohibitive surgical risk, valve-in-valve transcatheter mitral valve replacement was attempted but did not reduce the severity of the prosthetic paravalvular leak (PVL) severity. Subsequent percutaneous PVL closure with a ventricular septal defect occluder successfully reduced the PVL severity and led to significant clinical improvement.

## Introduction

Prosthetic paravalvular leak (PVL) is a complication that occurs in roughly 9% of patients after bioprosthetic mitral valve surgery.^1^ Valve dehiscence can lead to a particularly severe form of PVL that carries a high risk of hemodynamic collapse and mortality. Surgical repair of such a complication is the traditional approach, but it carries a high mortality and morbidity risk. While there is evidence to support percutaneous repair of PVL, clinical data and experience of percutaneous repair in bioprosthetic valve dehiscence remains scarce.^2,3,4,5,6,7^ The most common causes of PVL include endocarditis, sewing ring disruption, calcification, and scarring.^1^ Here we present our experience with a patient with mitral bioprosthetic valve dehiscence secondary to infective endocarditis who underwent a percutaneous mitral valve-in-valve and PVL repair.

## Case presentation

A 53-year-old male with a past medical history of intravenous drug use and infective endocarditis of the mitral valve presented with worsening shortness of breath 5 months after undergoing mitral valve replacement with a 31-mm Medtronic Mosaic bioprosthetic valve. His immediate postoperative transthoracic echocardiogram (TTE) had shown a normal functioning bioprosthesis. The patient reported intravenous (IV) drug use prior to this admission but blood cultures were negative. On TTE performed at our facility, he was found to have a rocking motion of the bioprosthetic mitral valve suggestive of valvular dehiscence. Shortly after presentation, the patient developed profound cardiogenic shock and was unresponsive to medical management and intra-aortic balloon pump. The etiology of the valvular dehiscence was thought to be related to prior endocarditis from his ongoing drug use, with one hospitalization for fever at an outside hospital after his bioprosthetic valve replacement.

**Figure 1:**
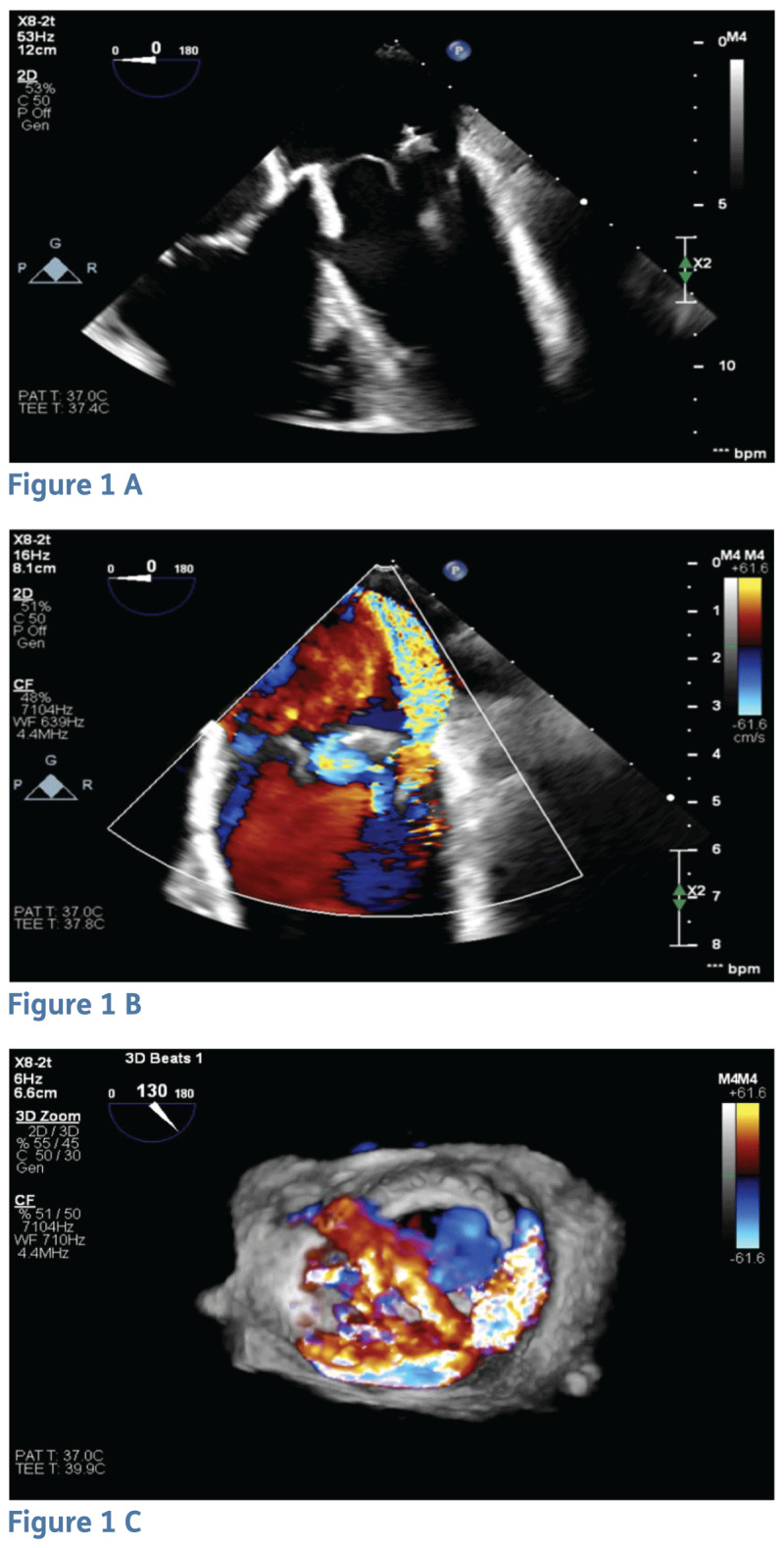
Transesophageal echocardiogram view of bioprosthetic mitral valve showing **(A)** valvular dehiscence, **(B)** color Doppler of severe paravalvular leak, and **(C)** 3-dimensional image.

An Impella CP was placed with stabilization of hemodynamics. Transesophageal echocardiogram (TEE) showed a crescentic shape of the dehiscence in the 5 to 7 o’clock region measuring approximately 1.6 cm in circumference by 0.6 cm in maximal width with severe paravalvular regurgitation (Figure 1). With multiorgan failure and ongoing IV drug use, his Society of Thoracic Surgeons score was 74.2%. In view of the large paravalvular regurgitation and marked prosthetic instability with rocking motion, we planned a salvage procedure with a mitral valve-in-valve (ViV) to stabilize the valve first and then proceed with PVL closure.

**Figure 2:**
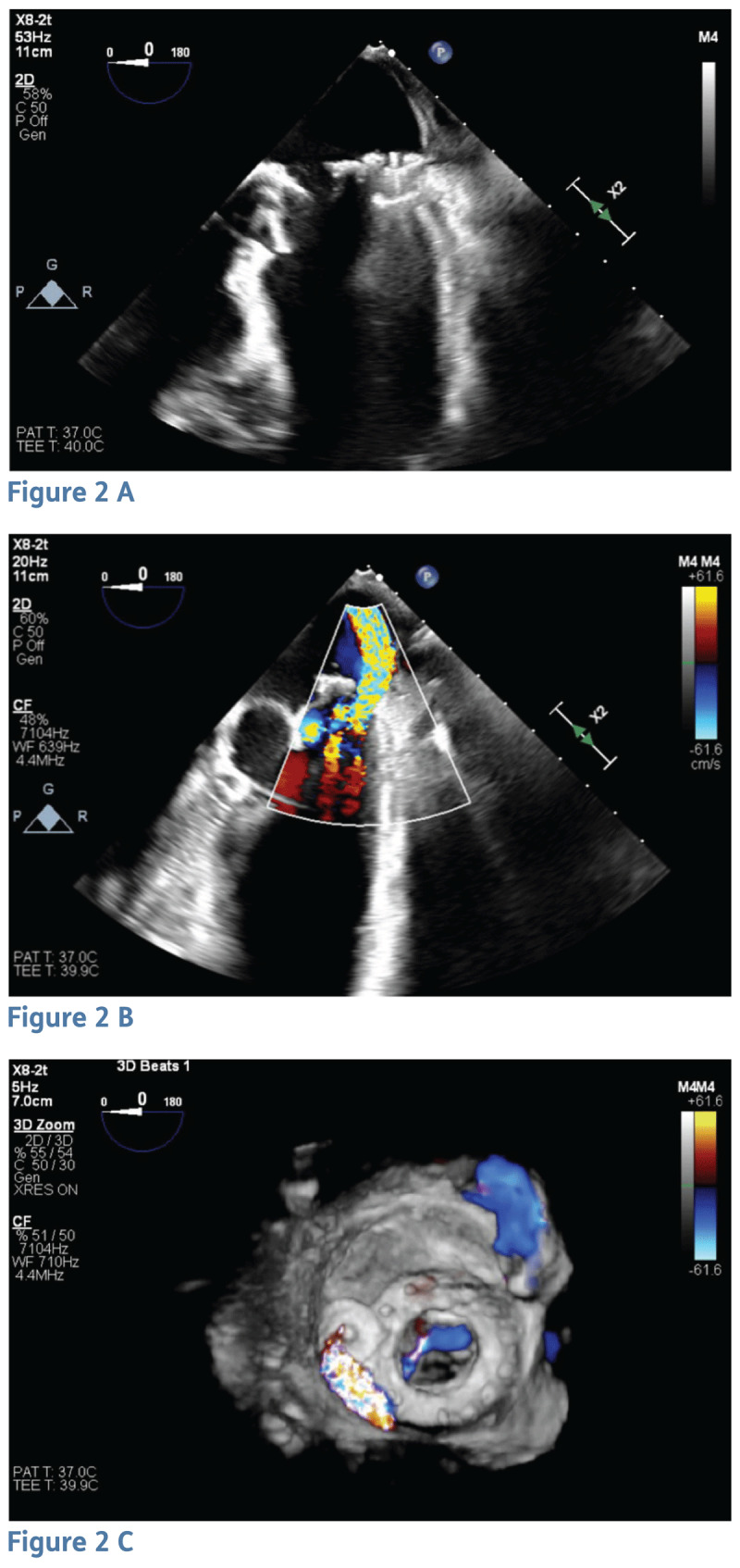
Transesophageal echocardiogram showing **(A)** septal occluder device in the lateral aspect of the bioprosthetic mitral valve, **(B)** color Doppler of modestly reduced paravalvular leak (PVL) after deploying septal occlude device, and **(C)** 3-dimensional image of reduced PVL.

The patient underwent a successful percutaneous transcatheter bioprosthetic mitral ViV replacement with a 29-mm Edwards SAPIEN 3 Ultra valve. Biological valve fracture of the 31-mm Mosaic valve (true inner diameter 26 mm) was attempted with a 28-mm TRUE balloon (Bard Vascular Inc.) but was unsuccessful despite achieving 18 atm of pressure, which resulted in rupturing the balloons twice. This was done via a transvenous route with transseptal puncture in the antegrade direction. Severe paravalvular regurgitation persisted. Next, an 8-mm ventricular septal occluder device was implanted to reduce regurgitation and stabilize the valve. Since the defect was crescentic in shape, we were unable to place two devices in the PVL despite attempting simultaneous deployment of both. Sequential deployments of the devices caused the first device to be dislodged. Therefore, we elected to use the single largest device that could be safely deployed in the defect with acceptable reduction in mitral regurgitation (MR) and without interfering with the mitral valve leaflets. As a result, valve stability was achieved, and there was a modest reduction in regurgitation (Figure 2), which led to significant clinical improvement.

The patient’s inotropic and vasopressor requirements and oxygenation improved, and the Impella CP was removed. There was no evidence of hemolysis, and kidney function improved. He was discharged 1 week later in stable condition with a plan to reassess MR and redo surgical valve replacement in 6 months if MR remained significant.

## Discussion

Severe prosthetic PVL is a challenging complication after mitral valve surgery, with a high risk of morbidity and mortality. Long-term survival from surgical management of PVL is estimated at 30% to 57.8%.^8^ Redo surgery is the traditional approach in such cases when surgical risk is acceptable. There is limited evidence in the literature for a percutaneous approach, and evidence and experience in valve dehiscence remains scarce. In addition, the literature on transcatheter PVL closure is varied as there are different definitions for procedural success, and some studies define “procedural success” as reduction by at least 1 degree of severity.

Here we present a case of mitral valve dehiscence and profound cardiogenic shock. The cause of dehiscence was likely a previous episode of endocarditis. Multiple blood cultures during current admission were negative, ruling out active infection. Our patient was at a prohibitive risk for surgical treatment and underwent successful percutaneous mitral ViV and deployment of a septal occluder device, which resulted in a reduction of PVL and hemodynamic stability. Our goal with transcatheter mitral ViV was to initially stabilize the bioprosthetic mitral valve, given the instability with its rocking motion, and also to reduce some of the PVL. This case shows that in such circumstances, percutaneous ViV and paravalvular plug closure might be a reasonable (albeit last resort) option. One of the challenges in our case was the inability to fracture the surgical valve swing ring despite multiple attempts with the largest balloons available. Despite this, we were still able to use an occluder device to plug the paravalvular area. Furthermore, complete resolution of MR is not always necessary to produce clinical improvement, as evident in our patient’s case. Even a modest reduction in the severity of PVL allowed the patient to be weaned off circulatory support and inotropes and to safely transition to discharge.
